# A five-lncRNA model predicting overall survival in gastric cancer compared with normal tissues

**DOI:** 10.18632/aging.203685

**Published:** 2021-11-09

**Authors:** Congbo Cai, Lei Yang, Xieyan Zhuang, Yi He, Kena Zhou

**Affiliations:** 1 Emergency Department of Yinzhou No. 2 Hospital, Ningbo 315000, Zhejiang, China; 2 Gynecology Department of Mingzhou Hospital, Ningbo 315000, Zhejiang, China; 3 Gastroenterology Department of Ningbo No. 9 Hospital, Ningbo 315000, Zhejiang, China

**Keywords:** lncRNA, gastric cancer, prognostic model, TCGA, GTEx

## Abstract

Aims: In cancer research, normal tissues adjacent to the tumor are usually defined as controls to compare with tumor samples, in order to screen out cancer-related genes. Although there is no obvious difference in pathology between normal tissues adjacent to the tumor and healthy tissues, there are significant changes at the molecular level. We aim to explore more potential tumor biomarkers using healthy tissues as controls rather than normal tissues adjacent to the tumor.

Methods: Here we combine the Genotype-Tissue Expression project and The Cancer Genome Atlas for differential gene analysis. Gene Ontology and Kyoto Encyclopedia of Genes and Genomes analyses were applied in order to predict the biological effects of related lncRNAs.

Results: We established a 5-lncRNA prognosis model with an AUC value of 0.815. Pathway analysis indicated that 5-lncRNA mainly affected tissue carcinogenesis through PI3K-AKT signaling pathway, Focal adhesion, MAPK signaling pathway.

Conclusion: The 5-lncRNA prognostic model we set up is more conducive to assess the overall survival time of gastric cancer patients.

## INTRODUCTION

Gastric cancer (GC) is a fatal disease ranking the third cause leading to death in tumors, which is a serious public health problem [1]. The molecular heterogeneity of cancer is a main factor that determines the clinical outcome. In cancer research, histological normality also implies molecular normality. However, this assumption cannot be applied to normal tissues adjacent to tumors (NAT) [2]. Although there is no specific pathological change in NAT, it has changed at the molecular level [3]. Compared with healthy tissues, NAT has molecular changes already, such as transcription and epigenetic aberrations [4], allele imbalances and changes in telomere length [5]. In addition, the tumor microenvironment will also promote cell cancerization [6]. Therefore, it is difficult to obtain accurate differential genes, and easy to miss some important genes when taking NAT as a healthy sample [7].

Here, we introduce the Genotype-Tissue Expression (GTEx) project: the data comes from the autopsy samples of 714 healthy donors, covering the information of genotype, gene expression, histology, and clinical data in 53 organizations [8, 9]. This project provides a new perspective for studying the genetic variation of tumors and changes in cell biology [10]. The Cancer Genome Atlas (TCGA), a landmark cancer genomics program, molecularly characterized over 20,000 primary cancer and matched normal samples spanning 33 cancer types.

Long non-coding RNA (lncRNA) has abnormal expression in GC [11, 12]. They promote GC cell proliferation, migration and inhibit cell apoptosis [13, 14]. Therefore, lncRNAs can be used as biomarkers for early detecting of GC. And a combination of multiple lncRNAs can increase the accuracy of prediction [15].

In this study, we integrated the GTEx and TCGA databases to compare the differential genes between healthy and cancer samples. Five lncRNAs were finally selected out to make a prognostic model. TCGA database verified that the prognostic model could predict overall survival (OS) in GC patients.

## RESULTS

### Patients characteristics

Original RNA-Seq expression and clinical data of healthy tissues from 191 donors were recruited on the GTEx website (https://gtexportal.org/) before January 28, 2020. Raw RNA-seq expression and clinical data of 375 GC patients were downloaded from the TCGA database (https://www.cancer.gov/) until January 31, 2020. All the characteristics of the healthy donors and GC patients are shown in Table 1.

**Table 1:** Summary clinical characteristics of patients.

**Characteristics**	**Normal group** **(GTEx n=191)**	**Cancer group** **(TCGA n=375)**
Age category		
<60/≥60/NA	156/35/0	112/259/4
Gender		
Male/ Female	110/81	241/134
Vital status		
Alive/ Dead	0/191	244/131
Race		
White/ Black/ Asian/ NA	NA	237/11/74/53
Death Circumstances		
D0/D1/D2/D3/D4	168/3/13/3/4	NA
Tumor stage		
I/ II/ III/ IV/ NA	NA	53/111/150/38/23
T stage		
T1/ T2/ T3/ T4/ NA	NA	19/80/168/100/8
M stage		
M0/ M1/ MX	NA	330/25/20
N stage		
N0/ N1 / N2/N3/NX	NA	111/97/75/74/18
Histologic grade		
G1/ G2/ G3/ GX	NA	10/137/219/9

Death Circumstances: D0: Ventilator Case; D1: Violent and fast death; D2: Fast death of natural causes; D3: Intermediate death; D4: Slow death; NA, NX, MX, GX: Clinical data are unknown.

### Selection of differential lncRNAs and establishment of the prognostic model

A total of 1641 differential genes were screened out, among which 886 were up-regulated (54.0%) and 755 were down-regulated (46.0%). 11 differential lncRNAs most relevant to the patient's OS were screened out according to the P value (P <0.001) (Table 2).

**Table 2:** lncRNA predictors by univariate cox analysis.

**LncRNA**	**HR**	**z**	**p-value**
LINC00919	1.570357	4.733196	2.21E-06*
AC010457.1	1.333	4.462278	8.11E-06*
MIR217HG	1.398173	3.899361	9.64E-05*
AL022316.1	0.804911	-3.73495	0.000188*
TRHDE-AS1	1.135597	3.70907	0.000208*
AP000695.1	1.290631	3.609454	0.000307*
AC107021.2	1.291236	3.569529	0.000358*
AP000695.2	1.2453	3.473397	0.000514*
AC005586.1	0.771899	-3.32525	0.000883*
AC037198.1	1.202342	3.320859	0.000897*
LINC02408	1.217608	3.318819	0.000904*

HR, Hazard ratio; *: P<0.001.

Multivariate cox analysis was performed on the obtained differential genes. A 5-lncRNA prognosis model was established with the highest AIC (Akaike Information Criterion) score of 1456.13 (Table 3).

**Table 3:** The detailed information of 5 lncRNAs for GC patients.

**LncRNA**	**Coef**	**Exp (coef)**	**Se (coef)**	**z**	**Pr (>|z|)**
AL022316.1	-0.23105	0.7937	0.059712	-3.86943	0.000109**
AC037198.1	0.143174	1.15393	0.055549	2.577431	0.009954*
AP000695.1	0.197343	1.218162	0.07356	2.682756	0.007302*
TRHDE-AS1	0.064235	1.066343	0.035917	1.788421	0.073708
LINC00919	0.453674	1.574085	0.098805	4.591625	4.40E-06***

Coef, coefficient; *: P<0.01; **: P<0.001; ***: P<0.0001.

**Figure 1:**
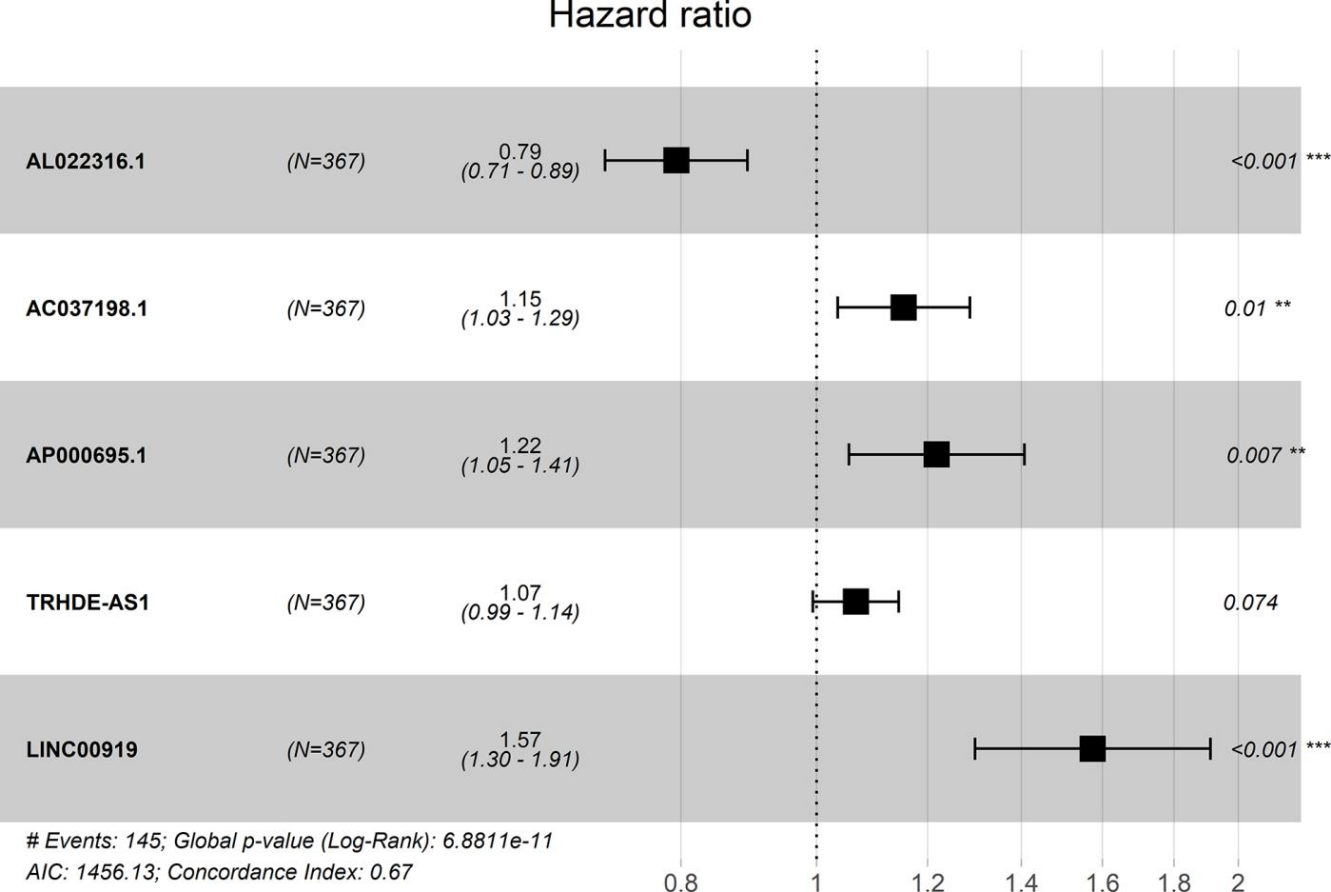
Forest plot for the association between five-lncRNA and risk value.

A forest plot showed the hazard ratio of five lncRNAs. The P value of this model was 6.8811E-11 and the C-index was 0.67 (Figure 1).

### Evaluation on the prognostic model

**Figure 2:**
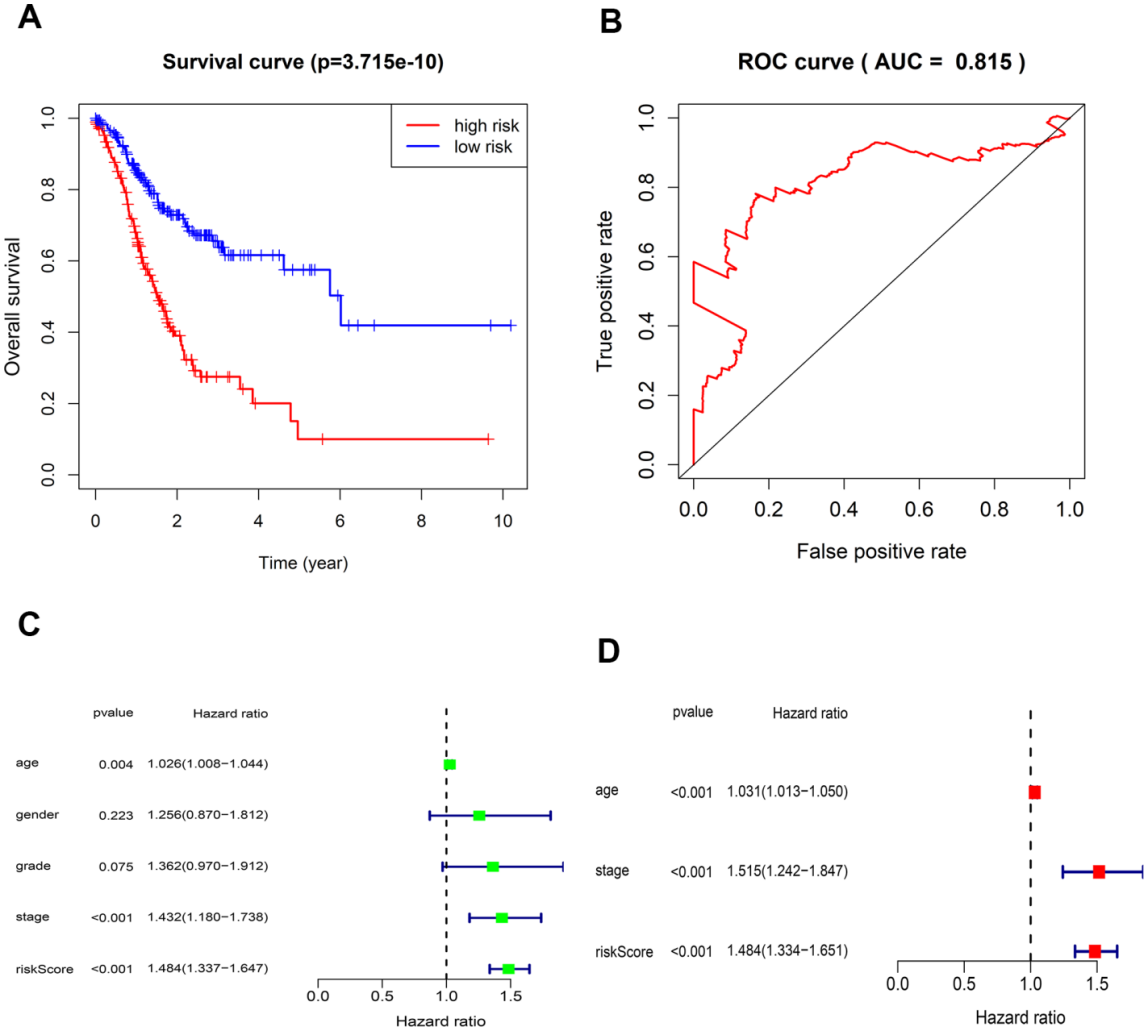
**Kaplan-Meier survival and ROC curves for the five-lncRNA in TCGA.** (**A**) The different OS between the high- and low-risk groups were determined by the log-rank test (p = 3.715e-10). (**B**) ROC curve for predicting 5-year survival with an AUC of 0.815. (**C**) Univariate Cox analysis evaluated the independent prognostic value of traditional clinical features and risk scores for OS in GC from TCGA. (**D**) Multivariate Cox analysis evaluates the independent prognostic value of traditional clinical features and risk scores for OS in GC.

The risk score of each patient can be calculated based on the expression level of 5-lncRNA and the coefficient value. The risk score formula is: (-0.23105×expression level of AL022316.1) + (0.143174×expression level of AC037198.1) + (0.197343×expression level of AP000695.1) + (0.064235×expression level of TRHDE-AS1) + (0.453674×expression level of LINC00919). All 375 patients were divided into high- and low-risk group according to the median risk score of 1.027. K-M curve suggested that the OS in the high-risk group was significantly lower than that in the low-risk group (P <0.001). The 5-year survival rate was 0.1 ± 0.058 (95% CI: 0.033-0.309) in high-risk group, and 0.575 ± 0.061 (95% CI: 0.467-0.708) in low-risk group (Figure 2A).

The AUC value of the 5-lncRNA prognosis model is 0.815, implying high accuracy and specificity (Figure 2B). The C-index value is 0.6664 (95% CI: 61.9-71.3%, P <3.563102E-12), which further proves the accuracy of the model. Univariate analysis showed that the risk score was significantly related to OS. Other variables like age and Stage were also associated with OS (Figure 2C). Multivariate analysis showed that the 5-lncRNA model was an independent prognostic factor in GC (Figure 2D).

**Figure 3:**
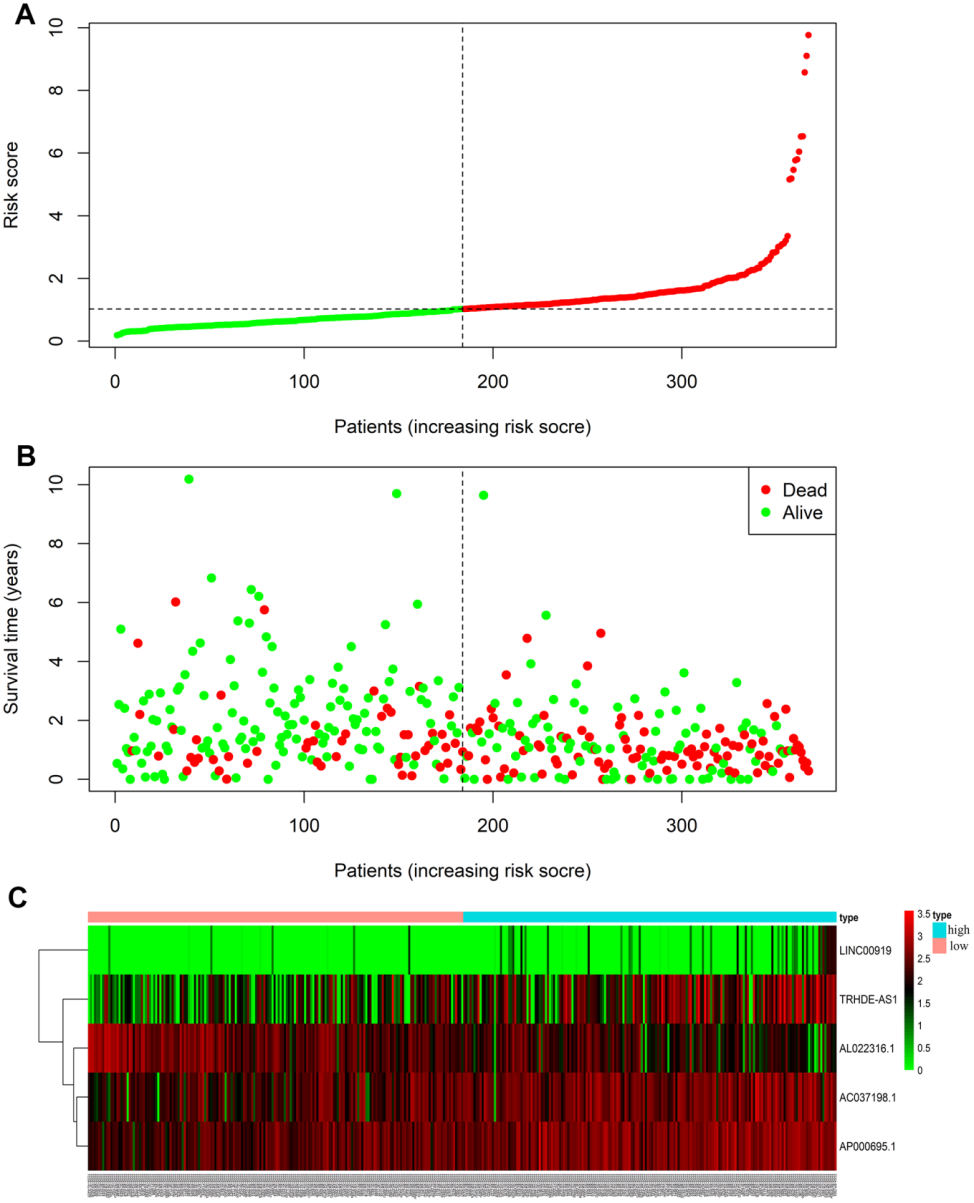
**LncRNA predictive risk-score analysis of GC patients in TCGA database (The black dotted line represents the median signature cut-off dividing patients into low- and high-risk groups).** (**A**) LncRNA risk score distribution in low- and high-risk groups (The green dots represent the low-risk patients, and red means the high-risk group). (**B**) The survival status and time of GC patients in low- and high-risk groups (The green dots represent alive, and red means dead). (**C**) Heatmap of the five-lncRNA expression profiles in GC patients.

Risks scores were sorted in order (Figure 3A), and we found that survival time of patients decreased and mortality rate increased as the risk value increased (Figure 3B). Moreover, expressions of the 4 lncRNAs (AC037198.1; AP000695.1; TRHDE-AS1; LINC00919) were negative to the risk score. While the expression of lncRNA (AL022316.1) was positive to the risk score.

### Biological function analyses of lncRNA

**Figure 4:**
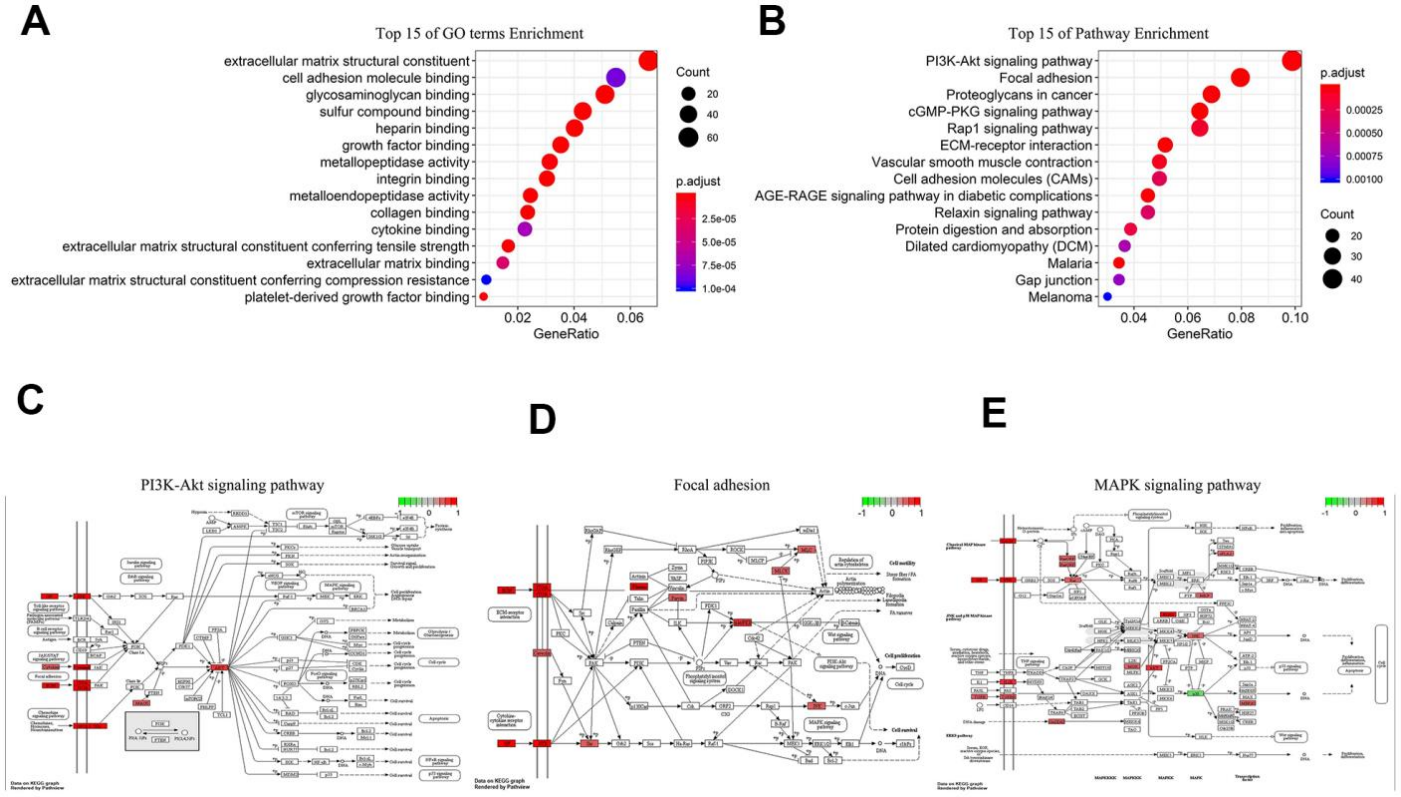
**Biological analysis of 5-lncRNA (Green represents low expression, Red means high expression).** (**A**) Top 15 enrichment terms in GO. (**B**) Top 15 pathways in KEGG. (**C**) PI3K-AKT signaling pathway. (**D**) Focal adhesion. (**E**) MAPK signaling pathway.

We performed Gene Ontology (GO) and Kyoto Encyclopedia of Genes and Genomes (KEGG) analyses on 1197 co-expression proteins of 5-lncRNA. GO illustrated that the 5-lncRNA prognosis model had 55 GO terms, and the first 15 were shown in Figure 4A, mainly containing extracellular matrix structural constituent and biomolecules binding. The 5 lncRNAs act on 36 major pathways, the most 15 pathways were shown in Figure 4B, mainly including PI3K-AKT signaling pathway (Figure 4C), Focal adhesion (Figure 4D), MAPK signaling pathway (Figure 4E). These 5 lncRNAs affect the progress of GC through the high expression of related signaling pathway proteins.

### Immune microenvironment with different risk values

CIBERSORT method and LM22 gene matrix were combined to analyze immune microenvironment.

**Figure 5:**
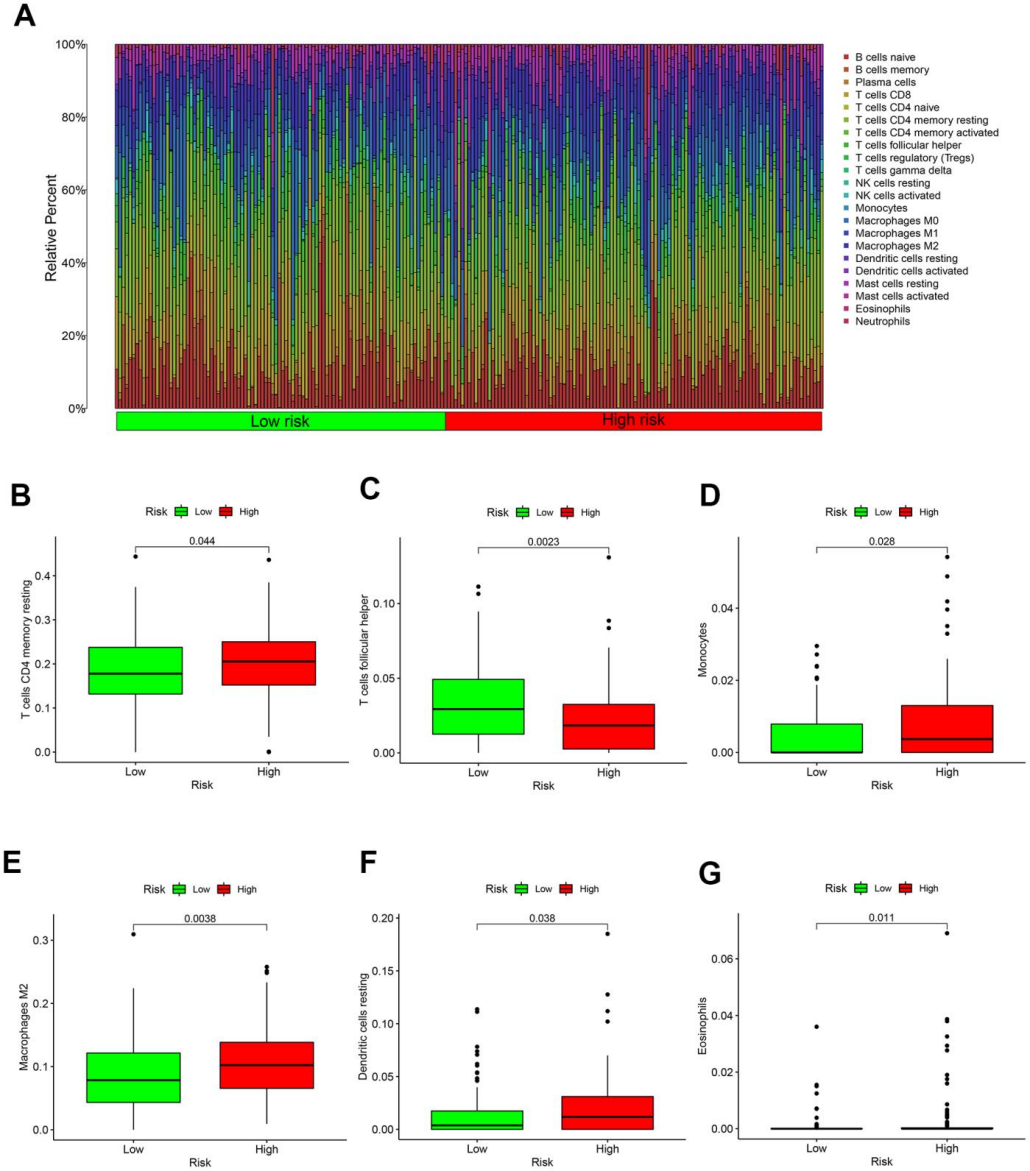
**Immune microenvironment in different risk groups.** (**A**) The relative proportion of 22 kinds of immune cell infiltration in high- and low-risk groups. (**B**) Box plot showing the expression of T cells CD4 memory resting in low- and high-risk groups. (**C**) Box plot showing the expression of T cells follicular helper in low- and high-risk groups. (**D**) Box plot showing the expression of Monocytes in low- and high-risk groups. (**E**) Box plot showing the expression of Macrophages in low- and high-risk groups. (**F**) Box plot showing the expression of Dendritic cells resting in low- and high-risk groups. (**G**) Box plot showing the expression of Eosinophils in low- and high-risk groups.

And the result of 22 immune cells in GC from the TCGA were exhibited in Figure 5A. Specific immune cells in high-risk patients are activated, such as T cells CD4 memory resting, Monocytes, Macrophages, Dendritic cells resting and Eosinophils (Figure 5B, 5D–5G). Only T cell follicular helper immune cells were highly expressed in the low-risk group (Figure 5C).

## DISCUSSION

LncRNA plays an important role in the occurrence and progression of GC [16, 17]. Multiple lncRNA prognostic models can evaluate the OS [15]. In this study, we screened out 11 lncRNAs most relevant to the OS in GC, and finally a brand-new prognostic model made up of 5-lncRNA was established after multivariate cox analysis. This prognostic model can evaluate the OS of GC patients precisely, with an AUC value of 0.815.

Many cancer studies currently use the TCGA database, and the exploration of cancer-related biomarkers is often based on the comparison between NAT and cancer samples [18]. Cai et al. set up a 9-lncRNA prognosis model (ADAMTS9-AS1, LINC01614, OVAAL, LINC02408, FLJ42969, LINC01446, CYMP-AS1, LINC01210, LINC01775) on the basis of TCGA database [19]. Cheng et al. promoted a 3-lncRNA prognostic model (CYP4A22-AS1, RP11-108M12.3AP000695.6) in GC [20]. Although their K-M analysis has good results, the AUC values are not ideal enough (AUC <0.8). In addition, both the two studies used NAT as a normal control group.

Aral et al. proposed that NAT was different from healthy tissues and tumor tissues [21]. And a recent research pointed out that NAT and tumor tissue have the same clonal origin, and many oncogenes are simultaneously expressed [22]. Furthermore, Nakashima et al. demonstrated that the molecular level of NAT tended to become cancerized [23]. Hence NAT can only be used as an intermediate state between healthy tissue and cancer tissue, which cannot be easily regarded as normal tissue [21]. A loss of many important lncRNAs will occur when using NAT as controls [7].

The GTEx database has sequencing of stomach tissues from healthy donors [10]. We are the first to use GTEx healthy samples as a control group to search for target genes, and finally obtained a brand new 5-lncRNA model. These 5 lncRNAs have not been reported in other articles. The prognosis model of 5-lncRNA has a higher AUC value than other models [19, 20]. We believe that in cancer research, healthy tissues are more suitable to serve as normal controls than NAT, and potential lncRNAs biomarkers related to the OS of GC patients can be obtained. In addition, the prognosis model of 5-lncRNA has a better accuracy than the original method (AUC value > 0.8).

With the emerging of deeper research, the role of some lncRNAs in GC has been revealed [24, 25]. However, functions of the vast majority of lncRNAs are still unclear. We performed GO and KEGG analysis on the five lncRNAs. GO analysis suggested that the main functions of this 5-lncRNA model were extracellular matrix structural constituent and biomolecules binding. KEGG showed that proteins co-expressed to the 5-lncRNA model was mainly enriched in PI3K-AKT signaling pathway, Focal adhesion, and MAPK signaling pathway. The top three signaling pathways happen to be important pathways in GC [26–29]. Therefore, we are more convinced that GTEx healthy samples can help us to discover potential lncRNAs to serve as novel biomarkers in GC.

Of course, there are some limitations about our study. First, healthy tissues and GC tissues were from different databases, and specimen collection and sequencing methods might be different. Although we used the original RNA-seq expression data and corrected them at the gene level, we still cannot completely eliminate the differences. Second, we lack information about the ethnicity of donors in GTEx database, so it is not possible to further clarify whether there are deviations between various populations. Third, although we have made multi-factor adjusted analysis on this model, age is a suspected confounding factor, which needs large scale of later researches to define exactly.

In conclusion, our study held the point that there was a significant difference between NAT and healthy tissue. And we proposed that healthy tissues were more suitable to be controls to obtain more potential biomarkers. The 5-lncRNA prognostic model can predict the survival risk of GC patients, but the detailed molecular mechanism might need to be evaluated by further research in the future. In the near future, we hope more biological experiments to be carried out to verify our results.

## MATERIALS AND METHODS

### Datasets collections

RNA-seq expression and clinical information of healthy gastric tissue were downloaded from GTEx database (https://gtexportal.org/). RNA-seq expression and clinical data of GC were downloaded from TCGA database (https://cancergenome.nih.gov/). All the above records are publicly open all over the world. There’s no need for admission from the Ethics Committee.

### The selection of differential lncRNAs

The "limma" package was used to correct the gene expression levels in healthy and GC tissues separately in R software. The "edgeR" package was used to compare the lncRNA expression levels between healthy and GC tissues, and differential lncRNAs were screened out with |log2(fold change)| ≥2 and P <0.05 as a criterion.

### Construction of GC-lncRNA risk score

According to the OS of 375 GC patients in the TCGA database, univariate cox analysis was performed on each differential gene. And differential genes most relevant to OS were selected according to P value (P <0.001). Then multivariate cox analysis on the selected differential genes was carried out to get the prognostic lncRNAs considering the AIC value. The coefficient values of these selected differential genes were also obtained. Afterwards, a prognosis model predicting OS in GC patients was established. The risk scoring formula of the model is: $“Risk\;score=\sum_{i=1}^{N}(Ei*Ci)”$, where N represents the prognostic lncRNA number, Ci represents the i-th lncRNA coefficient, and Ei represents the i-th lncRNA expression level. A forest map of the prognosis model was drawn in R software.

### Evaluation of the prognostic model

The risk value of each patient was calculated according to the scoring formula of the model. And patients were divided into high- and low-risk groups with the median risk value. Survival curves of the high- and low-risk groups were made by K-M method. Meanwhile the ROC curve of the prognostic model was drawn, and its AUC value was calculated. The consistency test on AUC value was performed using R package "survcomp." Univariate and multivariate cox regression analyses were to determine whether the prognostic model is an independent factor or not. Finally, the risk curve of the prognostic model was obtained based on the risk score ranking.

### Analysis on biological function of the prognostic lncRNAs

In order to predict the biological effects of related lncRNAs, we performed GO (Gene Ontology) and KEGG (Kyoto Encyclopedia of Genes and Genomes) analyses of the target 5 lncRNAs. We calculated the expression levels of all protein-coding genes (PCGs) corresponding to 5 lncRNAs through the R package "clusterProfiler." mRNAs with |Pearson correlation coefficient|> 0.40 and P <0.01 were considered to have co-expression.

### Immune microenvironment analysis

In order to evaluate the relative abundance of tumor-infiltrating immune cells in different risk groups, we used the CIBERSORT algorithm. This is a new calculation method developed by Newman et al., which uses a set of reference gene expression values (547 genes) to quantify the abundance of 22 immune cells [30]. Here we used CIBERSORT to evaluate the proportion of 22 immune cells in all GC patients from TCGA. And immune cell situation both in high- and low-risk groups were investigated.
